# Aesthetic results in children with single suture craniosynostosis: proposal for a modified Whitaker classification

**DOI:** 10.1007/s00381-022-05678-2

**Published:** 2022-09-22

**Authors:** Mahmoud Messerer, Rachel Cottier, Alberto Vandenbulcke, Amani Belouaer, Roy T. Daniel, Martin Broome, Giulia Cossu

**Affiliations:** 1 Department of Neurosurgery, University Hospital of Lausanne and University of Lausanne, Rue du Bugnon 46, 1010 Lausanne, Vaud, Switzerland; 2 Faculty of Biology and Medicine, University of Lausanne, Lausanne, Vaud, Switzerland; 3 Department of Maxillofacial Surgery, University Hospital of Lausanne and University of Lausanne, Lausanne, Vaud, Switzerland

**Keywords:** Craniosynostosis, Surgery, Whitaker classification, Aesthetic result

## Abstract

**Objective:**

Aesthetic assessment after surgery for non-syndromic single suture craniosynostosis (SSC) is crucial. Surgeons’ evaluation is generally based on Whitaker classification, while parental impression is generally neglected. The aim of this paper is to compare aesthetic perceptions of parents and surgeons after surgery for SSC, expressed by a 10-item questionnaire that complement Whitaker’s classification.

**Methods:**

The authors submitted a 10-item questionnaire integrating Whitaker’s classification in order to evaluate the degree of satisfaction, the detailed aesthetics results and the need for surgical revision, to surgeons and parents of a consecutive series of patients operated for SSC between January 2007 and December 2018. The results were collected blindly.

**Results:**

A total of 70 patients were included in the study. Scaphocephaly and trigonocephaly were the two most frequent craniosynostosis. Parents and surgeons general aesthetics evaluation and average rating for Whitaker’s classification were 1.86 vs 1.67 (*p* = 0.69) and 1.19 vs 1.1 (*p* = 0.45) respectively. Parents’ evaluation for scar perception and alopecia (*p* < 0.00001), the presence of bony crest (0.002), bony bump (*p* < 0.00001), or other bone irregularities (*p* = 0.02) are significantly worse when compared to surgeons’ perception.

**Conclusions:**

Parents seem to be more sensitive to the detection of some aesthetic anomalies and their opinion should not be neglected. The authors propose a modified Whitaker classification based on their results to better stratify the aesthetic outcome after surgery for SSC.

## Introduction

Craniosynostosis is a pathology affecting the membranous portions of the skull, causing premature fusion of one or multiple sutures. It can be an isolated finding or associated with genetic syndromes. The reported incidence of craniosynostosis range from 1:2000 to 1:2500 live births [1, 2]. Craniosynostosis cause characteristic skull deformities depending on the suture involved and the more severe cases, when untreated, can lead to increased intracranial pressure and consequent impaired neurodevelopment [3–5].

In non-syndromic single suture craniosynostosis (SSC), aesthetic concern is predominant. Cosmetic deformity includes cranial vault asymmetry (altered cephalic index), bumping or flattening, sutural ridings and dysmorphic facial features such as hypo or hypertelorism, and ear and ocular asymmetry up to strabismus. Surgical correction is the treatment of choice to normalise the cranial shape and prevent intracranial hypertension [6]. In the lasts decades, thanks to an improved awareness of the neurocognitive outcome and to the progress in surgical techniques, paediatric intensive care and anaesthesia, the advantage of early surgical correction was emphasised [7]. Aesthetics appearance of the face and skull receive the most attention in social interactions. Moreover, parental perception of aesthetics results may influence the development of child’s self-perception and self-confidence [8]. Finally, craniosynostosis correction may have a direct and indirect influence on neurocognitive outcomes.

Post-operative assessment and follow-up is fundamental to evaluate the surgical results. Most authors focused on surgeons’ evaluation using Whitaker scale [9] to asses aesthetics results. This is a scale ranging from I (no surgical revision needed) to IV (major craniofacial procedure needed). Parental contribution in Whitaker scale is not clear and this scale seems to lack in interrater reliability and predictive value [10]. Furthermore, parents and children carry the main stress and strains associated with the disease and laypersons perception of aesthetic results may differ from the surgeons [11–14].

To our knowledge few studies compare the aesthetic assessments of parents and surgeons [11, 14] and the purpose of this study is to compare aesthetic perceptions of parents and surgeons after surgery for SSC, expressed by a questionnaire that integrates Whitaker scale and incorporates parental assessment in outcome measures of surgery for SSC.

## Methods

We retrospectively reviewed the clinical data of our surgical cohort of patients operated for an open correction of SSC at the University Hospital of Lausanne between January 2007 and December 2018. The study protocol was approved by our local ethics committee before starting the study. We included all the patients operated before the age of 24 months and with a minimum postoperative follow up of 2 years. Patients presenting with a syndromic craniosynostosis or another cerebral pathology were excluded.

Clinical and epidemiological data such as age at surgery, sex, type of craniosynostosis and associated dysmorphism were retrieved from the electronic medical records. Patients were all operated according to the same surgical technique by the senior surgeons (MM and MB). For patients with scaphocephaly we performed a vault remodelling according the Renier’s “H” craniotomy method [15]. It included an open calvarial reconstruction through a bi-coronal zigzag scalp incision, with a 6-cm strip craniectomy including the affected sagittal suture from the coronal suture to the inion. Then we achieved lateral strip osteotomies in the parietal bone bilaterally along the coronal and lambdoid sutures. For trigonocephaly and brachycephaly the affected suture was removed and the reshaping of the anterior half of the convexity was accomplished from the coronal suture to the orbital rim, which was removed and remodelled to advance the orbital rim. The latter was fixed with absorbable plates, in order to optimise the aesthetic result in the orbital headband region. Laterally the extent of the osteotomy reached the pterion and the curves were fashioned with lateral closing wedge osteotomies.

**Fig. 1:**
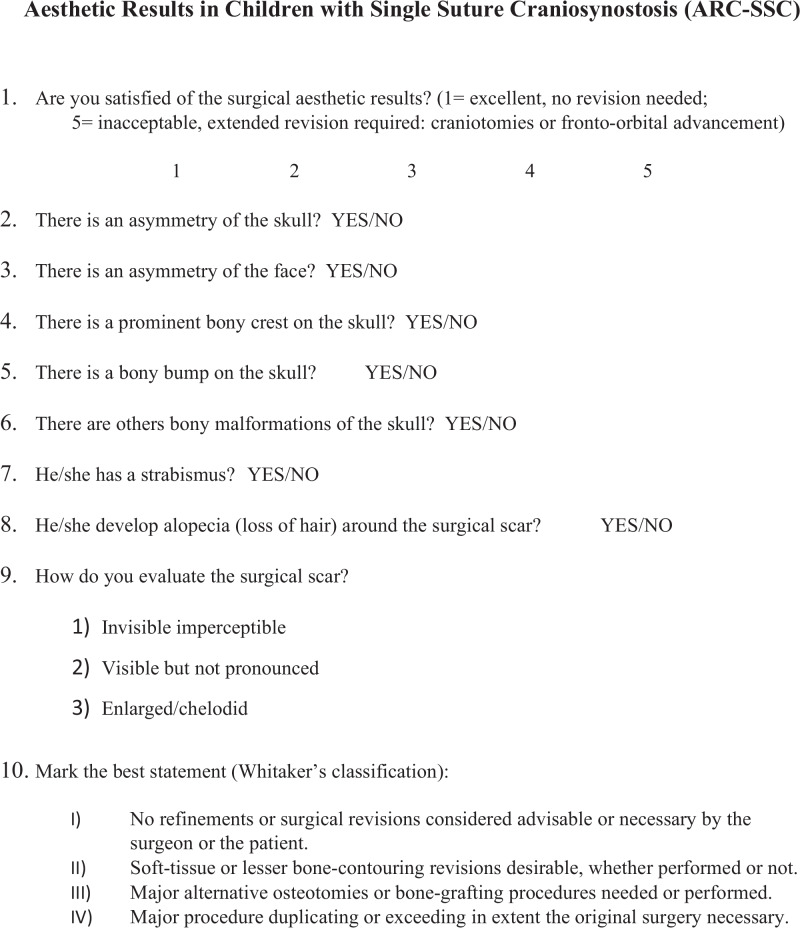
A 10-item questionnaire that we submitted to surgeons and parents of patients operated for a single-suture craniosynostosis

We drafted a 10-item questionnaire (Fig. 1) to integrate Whitaker’s classification in order to evaluate the degree of satisfaction, the aesthetics result and the need for surgical revision. The questionnaire included three scoring system: one for the general evaluation of the aesthetics results (ranging from 1: excellent result to 5: inacceptable, need surgical revision), one for the scar perception (corresponding to 1: imperceptible; 2: visible but not pronounced; 3: enlarged/chelodid) and one integrating Whitaker’s scale. Moreover, seven “yes or no” questions were used to determine the presence of skull and face symmetry, bony crest, alopecia, strabismus, skull deformities (bumps or others) (Fig. 1).

The questionnaire was submitted separately to the surgeon and the parents after the last follow up visit between January 2019 and December 2020: all the terms of the survey was explained to the parents during the outpatient clinic and then the parents received the questionnaire by mail after the clinic to be filled and returned.

The two groups were not aware of the counterpart’s responses and parental answers were collected by an external physician.

Continuous variables are presented as mean ± standard deviation (SD), and categorical variables as number and percentage. Univariate comparisons were performed with Fisher’s exact test and ANOVA test for categorical variables and Mann–Whitney *U* and Kruskal–Wallis test for continuous variables. Relationships between variables was evaluated through Spearman’s Rho. A *p* value < 0.05 was considered a significant difference. The analyses were performed using the STATA software version 15 (College Station, TX, StataCorp LP).

## Results

**Fig. 2:**
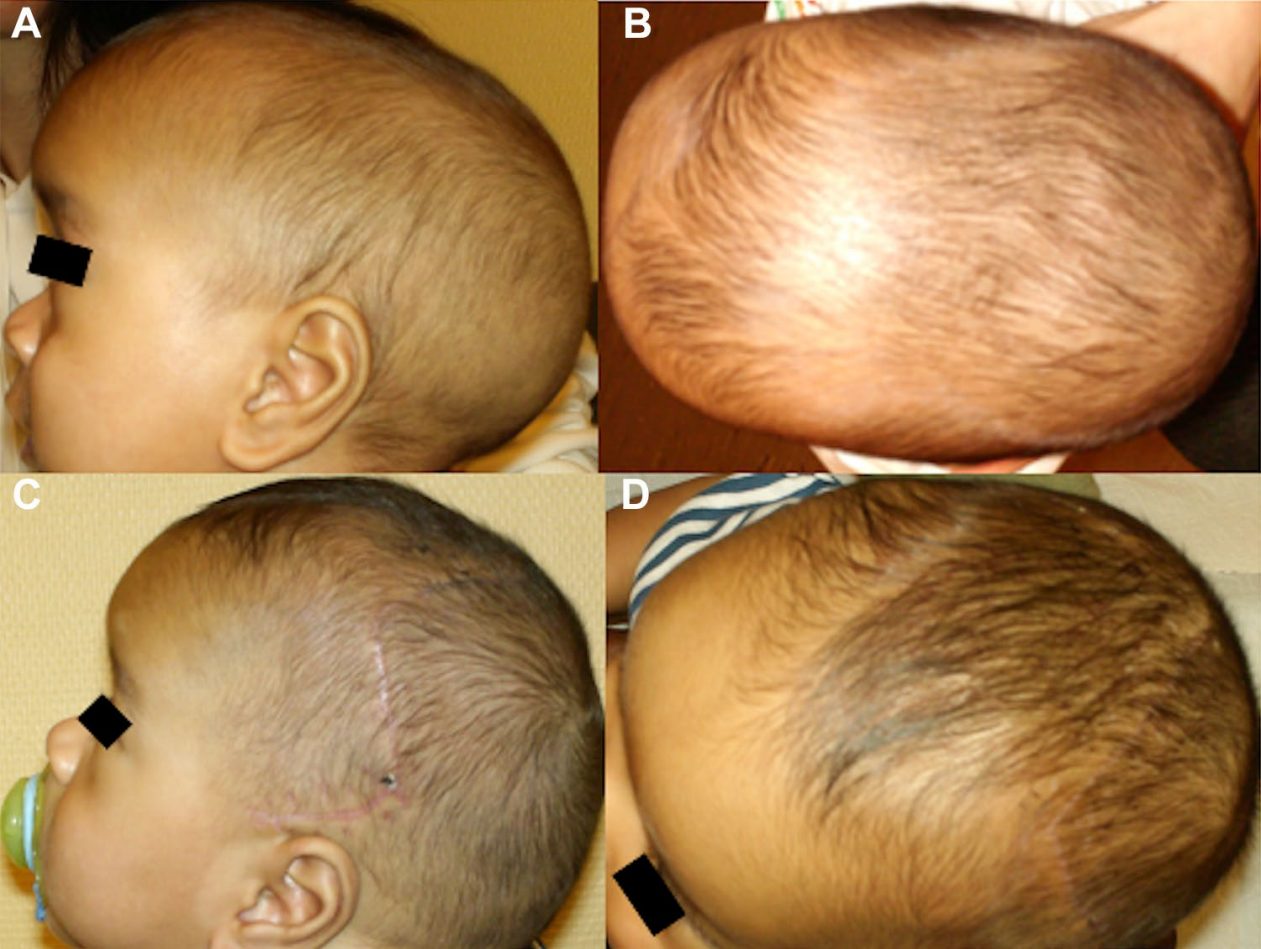
Pictures of a patient presenting a non-syndromic scaphocephaly before surgery (**A**, **B**) and 3 months after surgery (**C**, **D**)

**Fig. 3:**
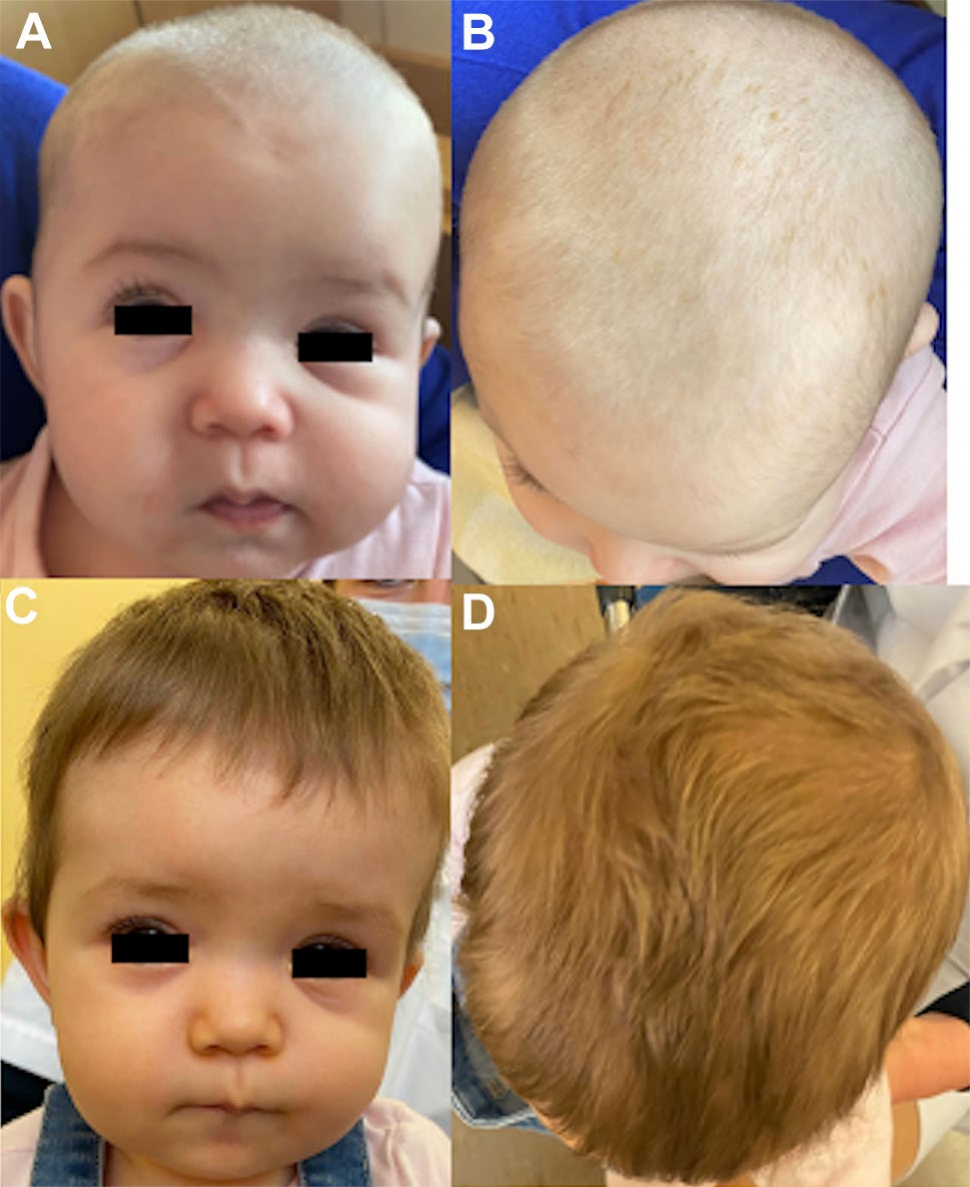
Pictures of a patient presenting a non-syndromic metopic synostosis at 3 months (**A**, **B**) and 5 years after surgery (**C**, **D**)

**Fig. 4:**
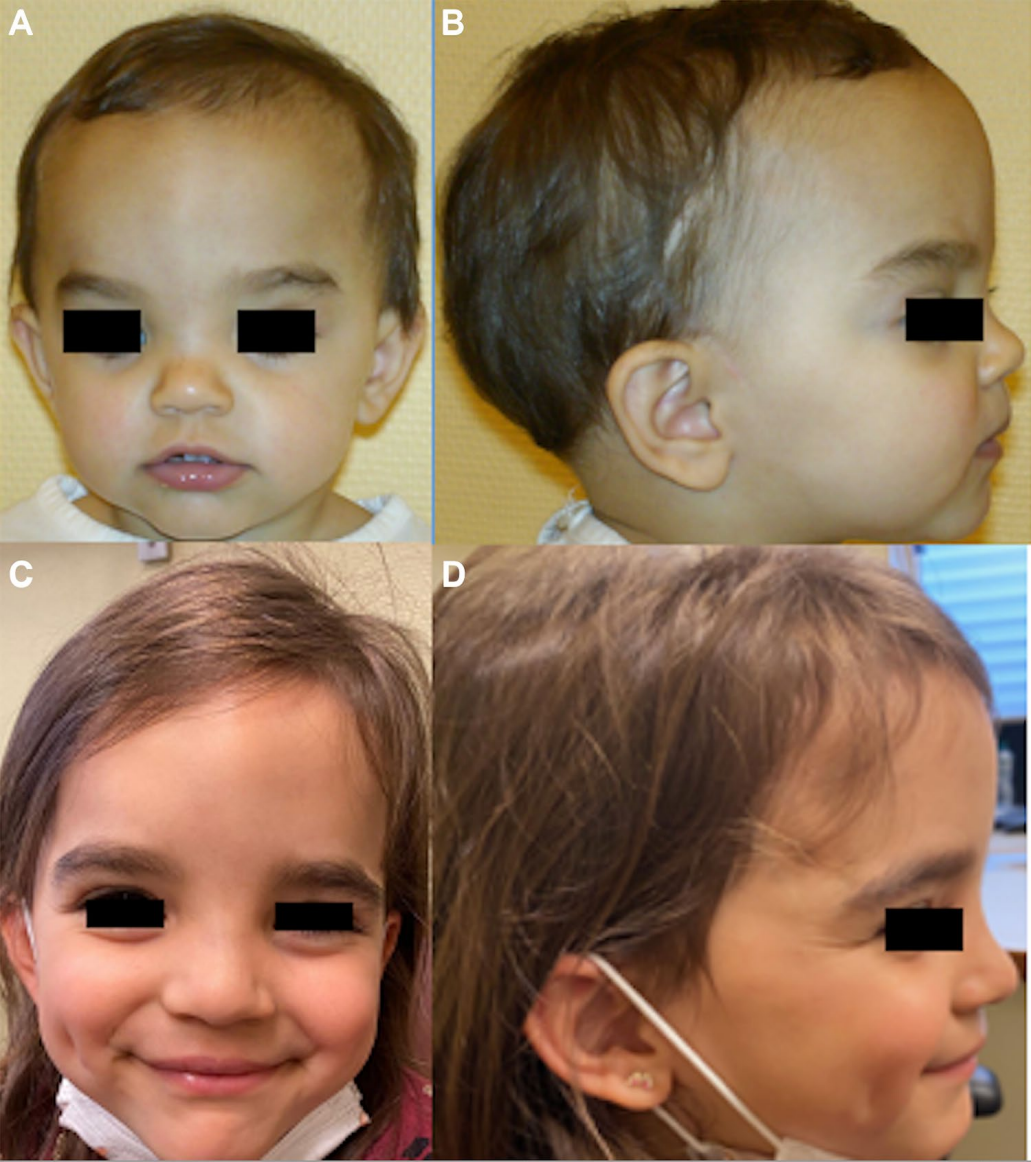
Pictures of a patient presenting a non-syndromic anterior plagiocephaly before surgery (**A**, **B**) and 6 months after surgery (**C**, **D**)

**Fig. 5:**
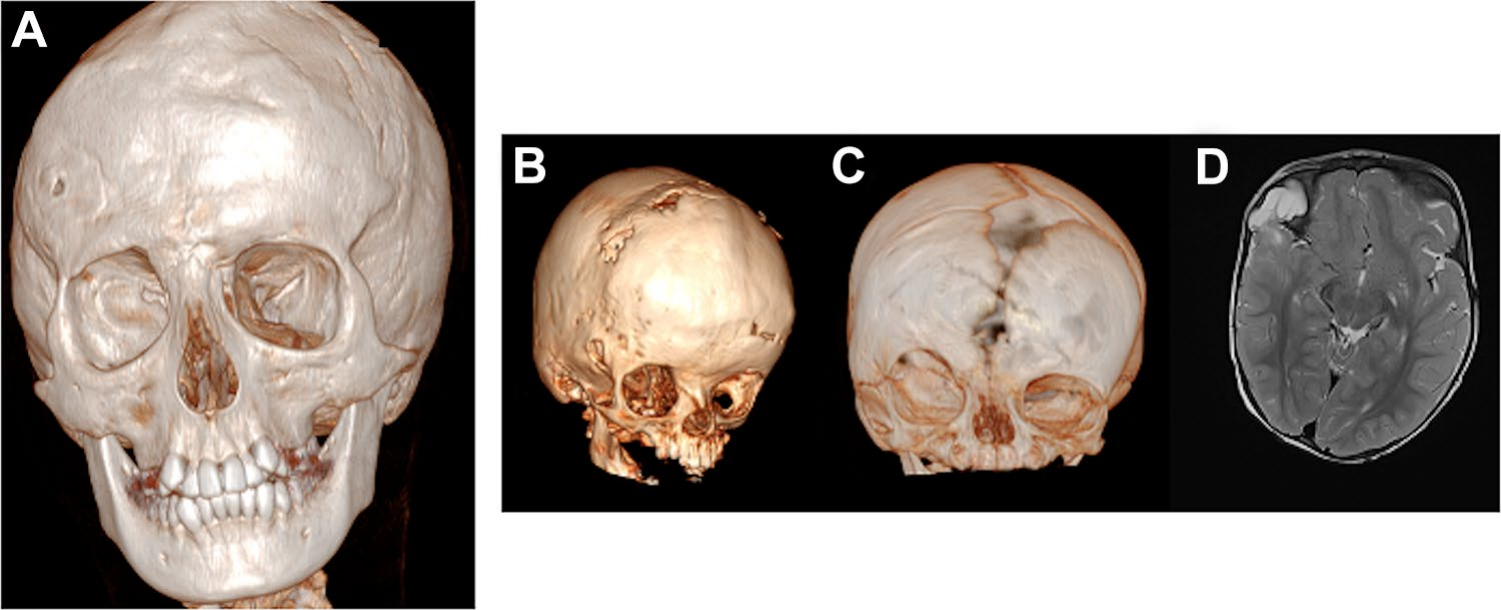
**A** A 3D reconstruction of a CT scan performed to evaluate the bony asymmetry after a surgical remodelling of an anterior plagiocephaly. A flattening of the right side of the frontal bone was confirmed. **B**, **C** 3D reconstructions of the postoperative results after plagiocephaly remodelling confirming a residual asymmetry of the frontal bone. **D** An axial T2-weighted MRI confirming the presence of an encephalocele, secondary to a dural tear accidentally performed during the first surgery. A second surgery performed 2 years after the first one allowed to address the frontal flattening and the treatment of the encephalocele

We identified 102 patients matching the inclusion criteria: 22 were lost at follow-up and 10 did not complete the questionnaire. A total of 70 patients were included in the study: 44 males and 26 females. Clinical and epidemiological data are reported in Table 1. Average age at surgery and at last follow up were 7 months (range 2–14 months, SD ± 2.33) and 7.2 years (range 2–14 years, SD ± 3.61) respectively. Scaphocephaly and trigonocephaly were the two most frequent craniosynostosis, accounting for 52.9% and 31.4% of cases of SCC respectively (Figs. 2 and 3). Plagiocephaly and brachycephaly accounted for 12.9% and 2.8% of cases respectively (Table 1) (Fig. 4). Three patients underwent revision surgery during follow up (4.3%): one patient presented a plagiocephaly and an orbito-frontal prosthesis was used to improve the surgical result 10 years after the primary surgery. The aesthetic result was not satisfying after the first surgery both for surgeons and parents but the family refused a craniotomy with secondary orbital advancement and a consensus was obtained to superpose a prosthesis. The management was multidisciplinary and the second surgery was performed by a team of neurosurgeon and maxillo-facial surgeon. A CT scan was performed to evaluate the bony asymmetry before the second surgery (Fig. 5A). The second patient presented a plagiocephaly: the surgical correction was complicated by a dural tear and the patient developed an encephalocele that was reoperated 2 years after the first procedure. This second surgery also addressed a minor flattening of the frontal bone. There was a discordance between parental perception and medical evaluation as the parents did only consider the aesthetic problem (Fig. 5B–D). The third patient presented a scaphocephaly and he recurred 11 months after surgery. Parents and surgeons agreed on the need of surgical revision (Whitaker class 4 and 3 for parents and surgeons respectively).

**Table 1:** Summary of clinical, epidemiological characteristic and surgical data for the cohort of patients operated for non-syndromic single suture craniosynostosis

**Characteristic**	**No (%; SD)**
Sex	
Female	26 (37%)
Male	44 (63%)
Mean age at surgery (months)	7 months (± 2.33 months)
Mean age at last follow-up (years)	7.16 years (± 3.61 years)
Sutures affected:	
Scaphocephaly	37 (52.9%)
Trigonocephaly	22 (31.4%)
Plagiocephaly	9 (12.9%)
Brachycephaly	2 (2.8%)
Postoperative complications:	5 (6.9%)
Haemorrhagic/anaemia	3 (4.2%)
Encephalocele (for dural tear)	1 (1.4%)
Thrombo-embolic	1 (1.4%)
Surgical revision	3 (4.2%)
Aesthetic problem	2 (2.8%)
Functional problem	1 (1.4%)

We had no major peroperative complications and minor postoperative complications are detailed in Table 1.

The questionnaires were performed at earliest 20 months after surgery, while the latest evaluation was performed 14 years after surgery. The results of our 10-item questionnaire are resumed in Table 2. Parents and surgeons general aesthetics evaluation and average rating for Whitaker’s classification were similar and both were in favour of a satisfying surgical outcome. No statistically significant difference was observed (*p* = 0.69 and *p* = 0.45 respectively). The details of Whitaker’s class are reported in Table 3: 85% of parents and 93% of surgeons reported that no refinements or surgical revisions were advisable (*p* = 0.71). We also stratified the results of Whitaker’s classification performed by surgeons and parents according to the timing after surgery and no correlation was found between the two variables (Spearman’s Rho, *p* = 0.5).

**Table 2:** The results of our 11-item questionnaire are here detailed and the differences between the evaluations performed by the parents and the surgeons are outlined

**11-item questionnaire**	**Parents no (%)**	**Surgeons no (%)**	***P*-value**
Surgical aesthetic results (mean)	1.86	1.67	0.68
-Skull asymmetry	17 (24.2%)	8 (11.4%)	0.08
-Face asymmetry	7 (10%)	8 (11.4%)	0.78
-Bony crest	15 (21.4%)	1 (1.4%)	***0***.***002***
-Bony bump	43 (61.4%)	13 (18.5%)	**< *0***.***00001***
-Others bony malformations	19 (27.1%)	6 (8.5%)	***0***.***017***
-Strabismus	13 (18.5%)	3 (4.2%)	***0***.***01***
-Alopecia	39 (55.7%)	1 (1.4%)	**< *0***.***00001***
Surgical scar evaluation (mean)	2.04	1.30	** < *0***.***00001***
Mean Whitaker (1–4)	1.19	1.11	0.45

**Table 3:** The aesthetic evaluations performed by the parents and the surgeons according to Whitaker’s classification are here detailed

**Whitaker’s class**	**Parents no (%)**	**Surgeons no (%)**	***P* values**
I: *No refinements or surgical revisions considered advisable or necessary by the surgeon or the patient*	58 (85.3%)	65 (92.9%)	0.71
II: *Soft-tissue or lesser bone-contouring revisions desirable*. *whether performed or not*	7 (10.3%)	3 (4.3%)	0.32
III: *Major alternative osteotomies or bone-grafting procedures needed or performed*	3 (4.4%)	1 (1.4%)	0.61
IV: *Major procedure duplicating or exceeding in extent the original surgery necessary*	0	1 (1.4%)	1

Concerning the others items, scar perception, the presence of bony crest, bony bump and alopecia are reported more frequently according to parents’ evaluation (*p* < 0.05). These evaluations were also stratified according to the timing from surgery but no statistically significant differences were found as the detection of these anomalies seemed to be independent from the distance from surgery in our surgical series.

Also, the strabismus and the presence of other bony irregularities are described more often by the parents (Table 2). However, specialised ophthalmological evaluation did not confirm the presence of strabismus.

## Discussion

Aesthetic result is one of the main outcome for surgical procedures performed for SSC and its regular and critical assessment is fundamental [6]. Whitaker’s classification is the most widely used to evaluate postoperative results and the need for further surgical correction [9]. The main criticism for this score is the lack of objectivity and the interobserver variability [10, 16]. Moreover it couples an aesthetic assessment with an operative decision and the need for revision may be under- or overestimated depending on surgeon’s personal opinion [10]. On the other hand, its strength is the simplicity. To introduce a second non-expert examiner may reduce the interobserver variability. The expectations regarding aesthetic outcome differ considerably between experts and non-experts [13] and surgeons may perceive the results as being better than parents do, especially for complex synostosis [14]. Only one study compared aesthetic outcomes for parents and medical teams using a scale similar to the Whitaker in a group of 87 children operated for sagittal synostosis. No statistical difference was observed between the two groups [11].

Our study is the first which integrated other specific outcomes to Whitaker’s classification to compare aesthetic perception between parents and surgeons in our surgical series of SSC. Parents spend much more times with their child and may focus more on the details, but the emotional component may overestimate the defects [16]. However, parents support their child during the development and their satisfaction may be conveyed to them and may influence their well-being, self-confidence and cognitive development [8]. Moreover, parental involvement in outcome evaluation is essential, as it could improve cooperation and trust with the medical team [11].

In our analysis, no statistical difference was observed when considering general aesthetics results, while parents show a major sensitivity for minor defects. However, the divergence in opinion regarding the scar appearance and the bony bumps did not affect Whitaker evaluation. It seems clear that the Whitaker classification is not sensitive enough to extensively evaluate the aesthetic outcome.

**Fig. 6:**
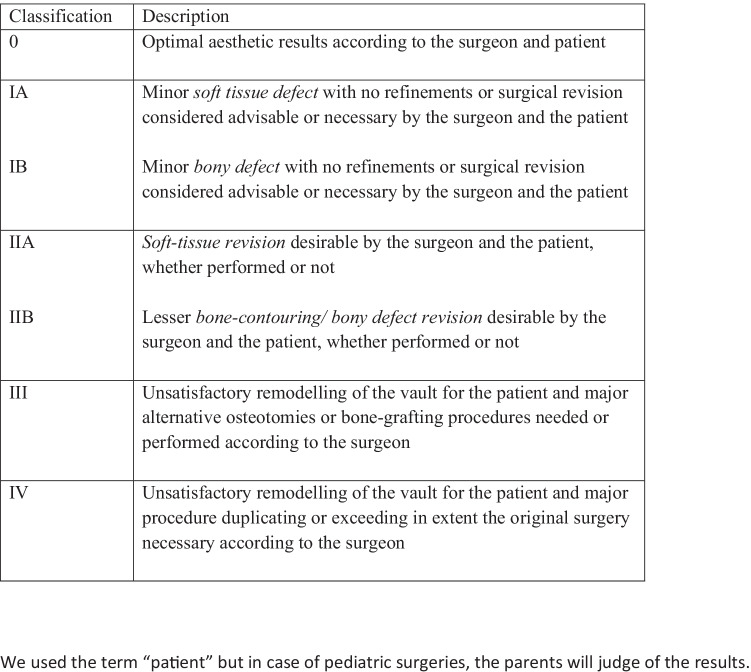
Our proposal of modified Whitaker classification. Class 0 is introduced to define patients with an excellent result. Class I is used to indicate the presence of minor defects, where no surgical revision was required. Classes I and II were separated into two categories: **A** indicated a soft tissue defect (including scar problem or alopecia), while **B** indicated a bone defect, namely bony crest and bumps

Moreover, coupling the aesthetic outcome with an operative decision seems to be a confounding factor. Some parents in our study underscore the results to avoid a surgical revision, as they desired an aesthetic improvement but without a proper surgery. In literature it is well-described the fact that late deformities may appear during the postoperative follow-up [17–19]. According to our analysis, we did not report any correlation between aesthetic problems and distance from surgery, but this may be attributed to the limited number of patients analysed. After the analysis of our data, to improve the aesthetic assessment and to increase the implication of parents in the decisional process, we proposed a revision of the classic Whitaker scale to the parents (Fig. 6). In our modified classification we added a grade 0 to include the patients with an optimal aesthetic result according to parents and surgeons. In the original Whitaker classification grade I include those patients in whom surgical refinement is not considered advisable or necessary, as synonym of good aesthetic results. In fact, this class include all patients with an optimal result as well as those with small defects not suitable for revision. By adding a grade 0, we could differentiate these two categories of patients. Furthermore, we introduced a subclass “A” and “B” to differentiate between soft-tissue and hard-tissue defect in grade I and II. In subclass A, we included scar problems and alopecia, while bony crests and bumps were included in subclass B. These were the items where a significant difference was detected between parental and surgeons’ evaluation according to our analysis: these sub-categories may require different surgical refinements, which can easily be addressed through mini-invasive or endoscopic techniques. Definitions of class III and IV were also simplified for parents’ understanding.

In our surgical series, we reported no major complications related to surgery and the rate of minor complication was similar to what reported in literature [19].

The main limitation of our study is the retrospective design with a questionnaire administered at last follow up and not regularly during the whole follow-up period. A larger prospective study should be performed to validate our proposed classification.

## Conclusions

The aim of craniosynostosis repair is to prevent aesthetic deformities and avoid neurocognitive problems. It seems obvious that parental opinion cannot be neglected and should be included in the outcome evaluation as minor postoperative defects such as bony crest and bumps and alopecia, were more frequently detected by the parents. The detection of these anomalies might however be dissociated from the willing of surgical revision. A modified Whitaker classification could improve patients’ stratification after surgery for SSC and help in distinguishing aesthetic outcomes from the need for surgical revision.
